# Preliminary exploration of dynamic changes in circulating mitochondrial DNA (ΔmtDNA) as a prognostic biomarker for acute ST-segment elevation myocardial infarction patients: a prospective observational study

**DOI:** 10.1186/s12872-026-06048-5

**Published:** 2026-06-05

**Authors:** Shipeng Tian, Lili Wang, Dongxiao Wang, Shiru Bai, Xian Wang

**Affiliations:** 1 Department of Cardiology, Hebei General Hospital, Shi Jia Zhuang City, He Bei 050051 China; 2 Hebei Key Laboratory of Precision Medicine and Translational Research on Cardiovascular Diseases, Hebei General Hospital, Shi Jia Zhuang City, He Bei 050051 China; 3 Department of Geriatric Cardiology, Hebei General Hospital, Shi Jia Zhuang City, He Bei 050051 China; 4 Hebei Provincial Clinical Research Center for Geriatric Diseases, Hebei General Hospital, Shi Jia Zhuang City, He Bei 050051 China

**Keywords:** Mitochondrial DNA, Acute ST-segment elevation myocardial infarction, Percutaneous coronary intervention, Major adverse cardiovascular events, Prognosis

## Abstract

**Background:**

Mitochondrial DNA (mtDNA) likely contributes to myocardial ischemia-reperfusion injury, yet the prognostic value of its dynamic changes (ΔmtDNA) for major adverse cardiovascular events (MACE) after percutaneous coronary intervention (PCI) in acute ST-segment elevation myocardial infarction (STEMI) patients remains unclear.

**Methods:**

This prospective study enrolled 236 STEMI patients undergoing emergency PCI. We measured serum mtDNA levels both before and after the procedure to calculate ΔmtDNA. Based on their ΔmtDNA values, patients were stratified into three groups: a Low ΔmtDNA group (T1, ΔmtDNA < 1.46), an Intermediate ΔmtDNA group (T2, 1.46 ≤ ΔmtDNA ≤ 3.62), and a High ΔmtDNA group (T3, ΔmtDNA > 3.62).

**Results:**

During a 1-year follow-up, 58 MACE cases occurred (incidence 24.5%). The Cox proportional hazards model indicated that each standard deviation increase in ΔmtDNA was associated with a 23.9% higher MACE risk (HR 1.239, 95% CI 1.161–1.321, *P* < 0.001). When ΔmtDNA was analyzed by tertiles, the risk displayed a dose-response relationship (P for trend < 0.001). Restricted cubic spline analysis supported a linear positive association (P for nonlinear = 0.263). Survival curves showed significantly different cumulative MACE risks among the ΔmtDNA groups (Log-rank *P* < 0.001). Subgroup analysis revealed a significantly stronger association between ΔmtDNA and MACE risk in patients treated with β-blockers or PCSK9 inhibitors (P for interaction < 0.05).

**Conclusions:**

This study demonstrates that ΔmtDNA is significantly associated with post-PCI MACE risk in STEMI patients, an association primarily driven by recurrent angina rather than hard endpoints such as mortality or myocardial infarction. These findings suggest that ΔmtDNA could serve as an exploratory biomarker to identify patients at elevated risk for ischemia-driven symptomatic events, but its clinical utility requires further validation through formal discrimination analyses and comparisons with established risk scores.

**Supplementary Information:**

The online version contains supplementary material available at 10.1186/s12872-026-06048-5.

## Introduction

Acute ST-segment elevation myocardial infarction (STEMI) is a life-threatening cardiovascular emergency [1]. Despite prompt vessel recanalization via emergency percutaneous coronary intervention (PCI), patients continue to face a substantial risk of long-term major adverse cardiovascular events (MACE), which presents a persistent clinical challenge [2]. Consequently, the development of biomarkers for the early and reliable identification of high-risk individuals is clinically important, as it would enable targeted secondary prevention and improve long-term outcomes.

Recent investigations into the pathophysiology of myocardial ischemia-reperfusion injury have established the central role of mitochondria. Acting as both the cellular powerhouse and a critical stress sensor, cells within the myocardium under ischemia-hypoxia sustain mitochondrial damage, which triggers the release of mitochondrial contents such as mitochondrial DNA (mtDNA) into the cytoplasm and circulation [3]. This circulating mtDNA acts as a damage-associated molecular pattern, activating innate immune pathways and amplifying local and systemic inflammatory responses [4]. These processes are closely linked to adverse cardiac remodeling and poor clinical outcomes.

Although basic research suggests a potential pathological role for mtDNA, its clinical translational value, particularly as a dynamic risk predictor, remains unclear. Previous clinical studies, largely confined to static measurements at single time points, have yielded inconsistent results and cannot readily distinguish the origin or progression of injury. Representing the quantitative change in circulating mtDNA during the perioperative period, ΔmtDNA more specifically reflects the net cellular damage and mitochondrial stress triggered by PCI and reperfusion itself, potentially serving as a promising dynamic predictor.

This study prospectively enrolled STEMI patients undergoing emergency PCI to determine whether ΔmtDNA predicts the risk of postoperative MACE.

## Materials and methods

### Study population

**Fig. 1:**
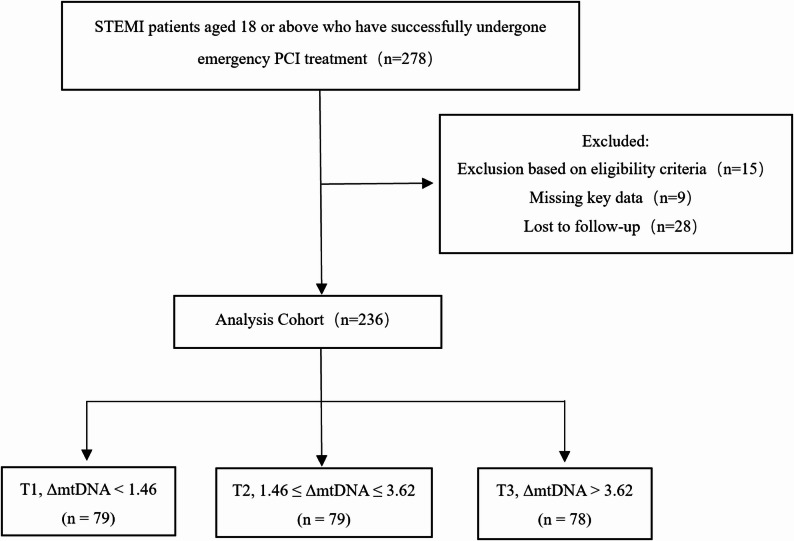
Flowchart of patient selection

This prospective observational study consecutively enrolled 288 hospitalized STEMI patients from the Department of Cardiology at Hebei Provincial People’s Hospital between March 2023 and February 2024. Fifteen patients were excluded based on predefined inclusion and exclusion criteria. A further nine patients were excluded due to missing critical clinical data, and an additional 28 patients were lost to follow-up. Consequently, 236 patients constituted the final study cohort for analysis, with the detailed screening process illustrated in Fig. 1.

#### Inclusion criteria

Patients were included if they: (1) were 18 years of age or older; (2) met the diagnostic criteria for acute ST-segment elevation myocardial infarction [5, 6]; (3) were hospitalized, provided informed consent for coronary intervention, and subsequently underwent successful emergency PCI; and (4) had no active chronic diseases, as detailed in the exclusion criteria.

#### Exclusion criteria

(1) Other cardiac disorders, including valvular heart disease, viral myocarditis, various cardiomyopathies (e.g., hereditary, alcoholic, or peripartum), pericardial diseases, immune-mediated cardiac disorders, congenital heart disease, and cor pulmonale, were excluded. These conditions intrinsically cause myocardial injury, heart failure, or arrhythmias, and their distinct pathophysiologies and prognoses compared to atherosclerotic STEMI could compromise the predictive value of ΔmtDNA for post-PCI MACE.

(2) Patients with severe infection at enrollment were also excluded; this was defined as an acute infection requiring intravenous antimicrobial therapy, meeting systemic inflammatory response syndrome criteria, or causing infection-related hemodynamic instability. Such infections can induce systemic inflammation and cellular damage, which significantly alter mitochondrial function and circulating mtDNA levels, thereby acting as potent confounders for the study outcomes [7].

(3) Active diseases: Active malignancy was defined as a current need for antitumor therapy (including chemotherapy, radiotherapy, targeted therapy, or immunotherapy), a diagnosis or treatment within the preceding six months, or the presence of active metastatic lesions; patients with a history of solid tumors considered clinically cured, such as those with no recurrence for over five years following surgery, were excluded.

Connective tissue diseases, including active-stage conditions such as systemic lupus erythematosus, rheumatoid arthritis, and vasculitis, require immunosuppressive therapy.

Other exclusions comprised poorly controlled severe chronic pulmonary diseases, including COPD at GOLD stages 3–4 or during an acute exacerbation, active infectious diseases like active pulmonary tuberculosis or viral hepatitis in a replicative phase, and poorly controlled hyperthyroidism or hypothyroidism.

These active conditions are frequently associated with a chronic systemic inflammatory state, immune dysfunction, or metabolic derangements. Such factors could independently influence both baseline mtDNA levels and cardiovascular outcomes, thereby introducing substantial and uncontrollable confounding bias.

(4) Bleeding disorders: including known coagulation dysfunction (e.g., hemophilia), active bleeding, or a history of major spontaneous bleeding.

Patients with these conditions face a substantially elevated risk of bleeding during the PCI perioperative period. The management of such patients, the occurrence of bleeding events, and the resulting clinical responses (e.g., transfusion, cessation of antithrombotic therapy) would profoundly confound the observation and attribution of MACE, especially ischemic endpoints.

### Data collection

The collected data comprised basic patient information (age, sex, smoking history), vital signs (systolic and diastolic blood pressure, heart rate), and comorbidities such as hypertension, coronary heart disease, cerebrovascular disease, and diabetes. We also recorded discharge medications, including ACEI/ARB/ARNI, β-blockers, PCSK9 inhibitors, and SGLT2i. Laboratory indicators encompassed Left ventricular ejection fraction (LVEF), hemoglobin, white blood cell count, admission Troponin T, creatine kinase isoenzyme, and low-density lipoprotein cholesterol. Coronary angiography features were documented, focusing on culprit vessel location and the presence of multivessel disease. Finally, procedure-related details included the use of mechanical circulatory support devices, the revascularization approach (drug-eluting stents versus plain balloon angioplasty/drug-coated balloons), and the vascular access route (radial or femoral artery).

### MtDNA collection and detection

Venous blood was collected in coagulant-containing vacuum tubes with inert separation gel one hour before PCI and again within 24 h post-procedure. All samples were immediately placed on ice at 4 °C and processed within two hours to minimize in vitro hemolysis. Serum was prepared via low-temperature multi-step differential centrifugation to remove platelet [8] interference: initial centrifugation at 800 rpm for 10 min at 4 °C was followed by transfer of the supernatant and a second spin at 1200 rpm, with a final centrifugation at 800 rpm to obtain serum, which was stored at -80 °C until analysis. Total DNA was extracted according to the protocol of the Circulating DNA Kit (Tingen, DP339). Mitochondrial DNA was quantified by SYBR Green-based real-time quantitative PCR (Tiangong, FP205) using the primer pair forward ATACCCATGGCCAACCTCCT and reverse GGGCCTTTGCGTAGTTGTAT. For normalization, the reference gene 18 S rRNA was simultaneously detected, and relative mtDNA levels were calculated via the ΔCt method [9]. The ΔCt for each sample was calculated as ΔCt = Ct(mtDNA) − Ct(18 S rRNA). Circulating cell-free mtDNA abundance was subsequently expressed as a relative quantity using the 2^−ΔCt^ method. To quantify perioperative dynamics, ΔmtDNA was defined as the absolute difference between the postoperative and preoperative relative quantities: ΔmtDNA = postoperative relative mtDNA − preoperative relative mtDNA. A positive ΔmtDNA value therefore indicates an increase in circulating mtDNA levels after PCI relative to baseline.

### Study grouping

The relative mtDNA quantities derived above were used to calculate ΔmtDNA for each patient, defined as the difference between the postoperative and preoperative values (ΔmtDNA = postoperative relative mtDNA − preoperative relative mtDNA). Patients were subsequently stratified into three groups according to the tertiles of this difference: a Low ΔmtDNA group (T1, ΔmtDNA < 1.46), an Intermediate ΔmtDNA group (T2, 1.46 ≤ ΔmtDNA ≤ 3.62), and a High ΔmtDNA group (T3, ΔmtDNA > 3.62).

### Follow-up

All patients were followed up through electronic medical records, outpatient visits, and telephone calls. The primary endpoint was the occurrence of a major adverse cardiovascular event (MACE) after the procedure, defined as cardiac death, non-fatal myocardial infarction, target vessel revascularization, recurrent angina, severe arrhythmia, severe heart failure, or an acute cerebrovascular event. Follow-up concluded in March 2025.

### Statistical analysis

Statistical analyses were conducted with R software (version 4.4.1) and SPSS 25.0. Continuous variables are presented as median (interquartile range, P25–P75) and were compared across groups using the Kruskal-Wallis test. Categorical variables are expressed as frequency (percentage) and were compared via the χ² test or Fisher’s exact test. Patients were stratified into tertiles (T1–T3) based on ΔmtDNA levels. The association between ΔmtDNA—analyzed as both a continuous and categorical variable—and the risk of MACE was evaluated using Cox proportional hazards regression models, which provided hazard ratios (HR) with 95% confidence intervals. Multivariable models were built sequentially, adjusting for predefined covariates drawn from established STEMI prognostic factors: age, Killip classification, LVEF, and estimated glomerular filtration rate (eGFR). A restricted cubic spline analysis within the Cox model framework was employed to examine the dose-response relationship between ΔmtDNA and endpoint risk. Survival curves were plotted using the Kaplan-Meier method and compared with the log-rank test. Subgroup analyses and interaction tests were performed to assess whether the primary association was modified by key clinical factors. All P-values were two-sided, and statistical significance was set at *P* < 0.05.

## Results

This study enrolled 236 participants, predominantly male (180 cases, 76%), with a mean age of 60 ± 13 years. The median ΔmtDNA level was 2.40 (IQR: 0.97–4.38). During follow-up, 58 MACE occurred (incidence: 24.5%), which included 10 all-cause deaths (4.2%), 8 non-fatal myocardial infarctions (3.4%), 16 target vessel revascularizations (6.8%), 23 cases of recurrent angina (9.7%), and 1 ischemic stroke (0.4%). Five patients (2.1%) required mechanical circulatory support, specifically intra-aortic balloon pump (IABP) in all 5 cases, with 2 of these also receiving extracorporeal membrane oxygenation (ECMO); no patient received Impella support. Two of these patients died during hospitalization.

### Comparison of baseline characteristics

No statistically significant differences were observed among the three groups for age, sex, vital signs, smoking history, Killip classification, or most comorbidities (*P* > 0.05). Significant intergroup differences were, however, identified in the prevalence of chronic kidney disease and hypoalbuminemia (*P* < 0.05). Differences in medication therapy were also evident, with a significant variation in the utilization rate of β-blockers (*P* < 0.05). Laboratory analysis further indicated that the T3 group demonstrated more pronounced elevations in white blood cell count, urea, creatinine, troponin T, and CK-MB levels (all *P* < 0.05). The complete dataset is provided in Table 1.

**Table 1:** Baseline information about the study population

Variables	Total (*n* = 236)	Tertile 1 (<1.46) (*n* = 79)	Tertile 2 (1.46–3.62) (*n* = 79)	Tertile 3 (>3.62) (*n* = 78)	*P* value
Demographics					
Age, years	60 (51, 70)	61 (54,70)	59 (49,68)	60 (51,72)	0.425
Gender, n(%)					0.465
Male	180 (76)	64 (81)	59 (75)	57 (73)	
Female	56 (24)	15 (19)	20 (25)	21 (27)	
Smoke, n(%)					0.211
No	150 (64)	50 (63)	45 (57)	55 (71)	
Yes	86 (36)	29 (37)	34 (43)	23 (29)	
Vital signs					
SBP, mmHg	136 (119, 153)	140 (121,159)	133 (114,147)	135 (119,152)	0.191
DBP, mmHg	82 (70, 95)	83 (73,95)	79 (69,92)	81 (70,96)	0.538
HR, beats/min	83 (72, 93)	84 (76,93)	81 (67,90)	84 (72,96)	0.196
Clinical Presentation					
Killip class, n(%)					0.136
Killip class I	172 (73)	64 (81)	54 (68)	54 (69)	
Killip class ≥ II	64 (27)	15 (19)	25 (32)	24 (31)	
Comorbidities					
Hypertension, n(%)					0.468
No	98 (42)	35 (44)	35 (44)	28 (36)	
Yes	138 (58)	44 (56)	44 (56)	50 (64)	
CHD, n(%)					0.066
No	187 (79)	62 (78)	57 (72)	68 (87)	
Yes	49 (21)	17 (22)	22 (28)	10 (13)	
CVD, n(%)					0.368
No	204 (86)	71 (90)	65 (82)	68 (87)	
Yes	32 (14)	8 (10)	14 (18)	10 (13)	
DM, n(%)					0.874
No	164 (70)	55 (70)	57 (72)	52 (68)	
Yes	70 (30)	24 (30)	22 (28)	24 (32)	
Nephrosis, n(%)					0.014
No	222 (94)	77 (97)	77 (97)	68 (87)	
Yes	14 (6)	2 (3)	2 (3)	10 (13)	
Hyperlipidemia, n(%)					0.920
No	180 (76)	61 (77)	59 (75)	60 (77)	
Yes	56 (24)	18 (23)	20 (25)	18 (23)	
Hypoproteinemia, n(%)					< 0.001
No	220 (93)	79 (100)	76 (96)	65 (83)	
Yes	16 (7)	0 (0)	3 (4)	13 (17)	
Anaemia, n(%)					0.051
No	224 (95)	77 (97)	77 (97)	70 (90)	
Yes	12 (5)	2 (3)	2 (3)	8 (10)	
Medication use					
ACEI/ARB/ARNI, n(%)					0.768
No	110 (47)	39 (49)	37 (47)	34 (44)	
Yes	126 (53)	40 (51)	42 (53)	44 (56)	
β blocker, n(%)					0.040
No	50 (21)	10 (13)	23 (29)	17 (22)	
Yes	186 (79)	69 (87)	56 (71)	61 (78)	
PCSK9 Inhibitor, n(%)					0.528
No	81 (34)	25 (32)	31 (39)	25 (32)	
Yes	155 (66)	54 (68)	48 (61)	53 (68)	
SGLT2i, n(%)					0.222
No	174 (74)	55 (70)	56 (71)	63 (81)	
Yes	62 (26)	24 (30)	23 (29)	15 (19)	
Laboratory parameters					
LVEF, %	57 (51, 62)	57 (52,63)	57 (51,62)	56 (50,60)	0.370
Hemoglobin, g/L	144 (131, 156)	148 (136,158)	144 (133,156)	143 (129,156)	0.361
Leukocyte, 10^9^/L	9.57 (7.61, 11.15)	8.97 (6.46,10.53)	8.60 (7.56,10.84)	10.56 (9.02,12.52)	< 0.001
Platelet, 10^12^/L	220 (187, 266)	217 (168,244)	218 (189,265)	224 (191,286)	0.139
Albumin, g/L	41.3 (39.1, 44.2)	41.5 (39.0,43.8)	40.8 (39.2,44.1)	41.6 (39.6,44.2)	0.813
Urea, mmol/L	5.53 (4.55, 6.56)	5.22 (4.59,6.40)	4.81 (4.31,5.84)	6.22 (5.16,9.17)	< 0.001
Creatinine, µmol/L	70.7 (60.3, 84.8)	76.0 (65.5,84.9)	65.5 (57.6,76.4)	74.0 (60.6,94.7)	0.013
eGFR, mL⋅min^− 1^ ⋅1.73 m^2^	95.71 (80.66, 107.37)	94.71 (77.76,104.35)	100.04 (87.99,108.47)	94.65 (68.38,106.77)	0.087
Troponin T, ng/L	107 (30, 341)	31 (30,143)	81 (30,246)	263 (96,882)	< 0.001
CK-MB, U/L	21.7 (11.8, 43.9)	16.8 (11.5,37.2)	21.6 (11.9,39.9)	34.7 (12.0,74.2)	0.006
Uric acid, µmol/L	347.8 (297.3, 410.7)	342.5 (294.7,393.1)	348.0 (302.6,394.5)	352.1 (288.5,420.5)	0.465
Total cholesterol, mmol/L	4.78 (3.97, 5.63)	4.84 (4.03,5.58)	5.09 (4.09,5.75)	4.53 (3.80,5.41)	0.256
Triglyceride, mmol/L	1.34 (0.97, 2.09)	1.29 (0.94,2.34)	1.39 (0.99,2.04)	1.38 (0.96,1.73)	0.991
LDL, mmol/L	3.12 (2.34, 3.72)	3.12 (2.33,3.72)	3.27 (2.49,3.71)	2.87 (2.26,3.72)	0.440
Lp a, mg/L	131.25 (60.33, 268.90)	105.80 (56.95,245.35)	125.80 (58.15,307.70)	153.45 (81.85,261.25)	0.305
Preoperative mtDNA	3.33 (1.99, 4.84)	1.77 (1.23,2.98)	3.48 (2.77,3.82)	6.20 (3.87,7.61)	< 0.001
Postoperative mtDNA	5.94 (2.64, 9.13)	1.88 (1.24,2.64)	5.66 (5.31,6.68)	14.53 (8.86,16.64)	< 0.001
Angiographic characteristics					
Culprit Vessel, n(%)					0.518
Left main or proximal LAD disease	99 (42)	37 (47)	30 (38)	32 (41)	
Other vessel disease	137 (58)	42 (53)	49 (62)	46 (59)	
Multivessel disease, n(%)					0.380
No	100 (42)	38 (48)	33 (42)	29 (37)	
Yes	136 (58)	41 (52)	46 (58)	49 (63)	
Procedural Details					
MCS, n(%)					0.623
No	231 (98)	76 (96)	78 (99)	77 (99)	
Yes	5 (2)	3 (4)	1 (1)	1 (1)	
Treatment, n(%)					0.325
DES	163 (69)	56 (71)	58 (73)	49 (63)	
POBA/DCB	73 (31)	23 (29)	21 (27)	29 (37)	
Access Site, n(%)					0.531
Radial access	198 (84)	69 (87)	66 (84)	63 (81)	
Femoral access	38 (16)	10 (13)	13 (16)	15 (19)	

The medications listed in the table are those prescribed at hospital discharge

*Abbreviations*: *SBP* systolic blood pressure, *DBP* diastolic blood pressure, *HR* heart rate, *CHD* Coronary Heart Disease, *CVD* Cerebrovascular Disease, *DM* Diabetes Mellitus, *ACEI* angiotensin-converting enzyme inhibitor, *ARB* angiotensin II receptor blocker, *ARNI* angiotensin receptor-neprilysin inhibitor, *SGLT2i* sodium-glucose cotransporter 2 Inhibitor, *LVEF* left ventricular ejection fraction, *eGFR* estimated glomerular filtration rate, *CK-MB* creatine kinase-MB, *LDL* low-density lipoprotein, *MCS* Mechanical Circulatory Support

### Cox regression analysis of ΔmtDNA and postoperative MACE risk

To assess the predictive value of ΔmtDNA for postoperative MACE risk, we employed a Cox proportional hazards regression model. Analyzed as a continuous variable per 1-SD increase, ΔmtDNA was independently associated with a 23.9% higher MACE risk after adjustment for age, renal function, cardiac function, and Killip classification (HR 1.239, 95% CI 1.161–1.321, *P* < 0.001; Model 2, Table 2), providing the principal support for our conclusion. In an exploratory model that included additional clinical variables (Model 3), this association persisted (HR 1.336). The interpretation of this result requires caution, however, owing to the number of covariates and the limited event count (Table S1). Stratification by ΔmtDNA tertiles revealed a dose-response relationship, with the intermediate (T2) and highest (T3) groups exhibiting multivariate-adjusted hazard ratios of 3.008 and 3.600, respectively, relative to the lowest group (T1) (P for trend < 0.001). This pattern corroborates the continuous variable analysis (Table S2). In summary, ΔmtDNA levels remained significantly associated with MACE risk after confounding adjustment in this dataset. In Model 3, which contained both ΔmtDNA and admission Troponin T, the correlation between these variables was moderate (Spearman’s *r* = 0.418, *P* < 0.001). Variance inflation factor (VIF) analysis showed no concerning collinearity (all VIF < 3). Nonetheless, given the modest number of MACE events and the potential for both markers to reflect related injury pathways, the estimates from this fully adjusted model warrant cautious interpretation.

**Table 2:** Multivariable Cox regression analysis of ΔmtDNA and postoperative MACE risk

ΔmtDNA	Crude mode		Model 1		Model 2	
	HR (95%CI)	*P* value	HR (95%CI)	*P* value	HR (95%CI)	*P* value
ΔmtDNA（per 1 SD elevation）	1.237 (1.161-1.317)	<0.001	1.237 (1.161-1.317)	<0.001	1.239 (1.161-1.321)	<0.001

Crude model was adjusted for none

Model 1 was adjusted for age

Model 2 was adjusted for model 1 + (estimated glomerular filtration rate, left ventricular ejection fraction, Killip class)

### Dose-response relationship analysis between ΔmtDNA and postoperative MACE risk

**Fig. 2:**
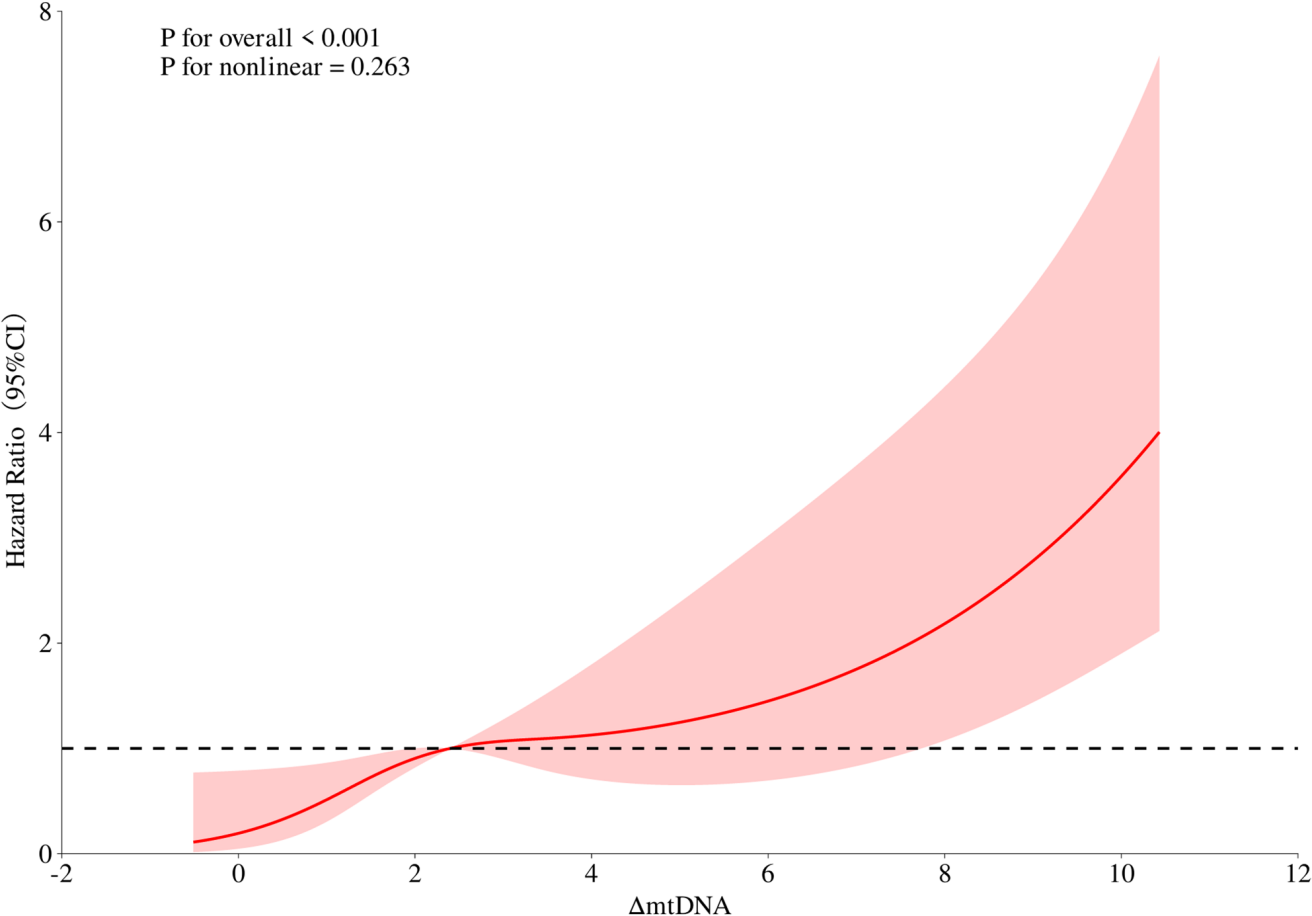
Linear relationship between ΔmtDNA and postoperative MACE risk, adjusted for age, estimated glomerular filtration rate, left ventricular ejection fraction, and Killip class. The solid red line represents the estimated association, and the pink shaded area indicates the corresponding 95% confidence interval

To characterize the dose-response relationship between ΔmtDNA and postoperative MACE risk, we employed restricted cubic spline models. Following multivariate adjustment, ΔmtDNA demonstrated a significant overall association with MACE risk (P for overall < 0.001). The relationship exhibited a linear trend (P for nonlinear = 0.263), as detailed in Fig. 2.

### Survival analysis of ΔmtDNA and postoperative MACE with Its component risks

**Fig. 3:**
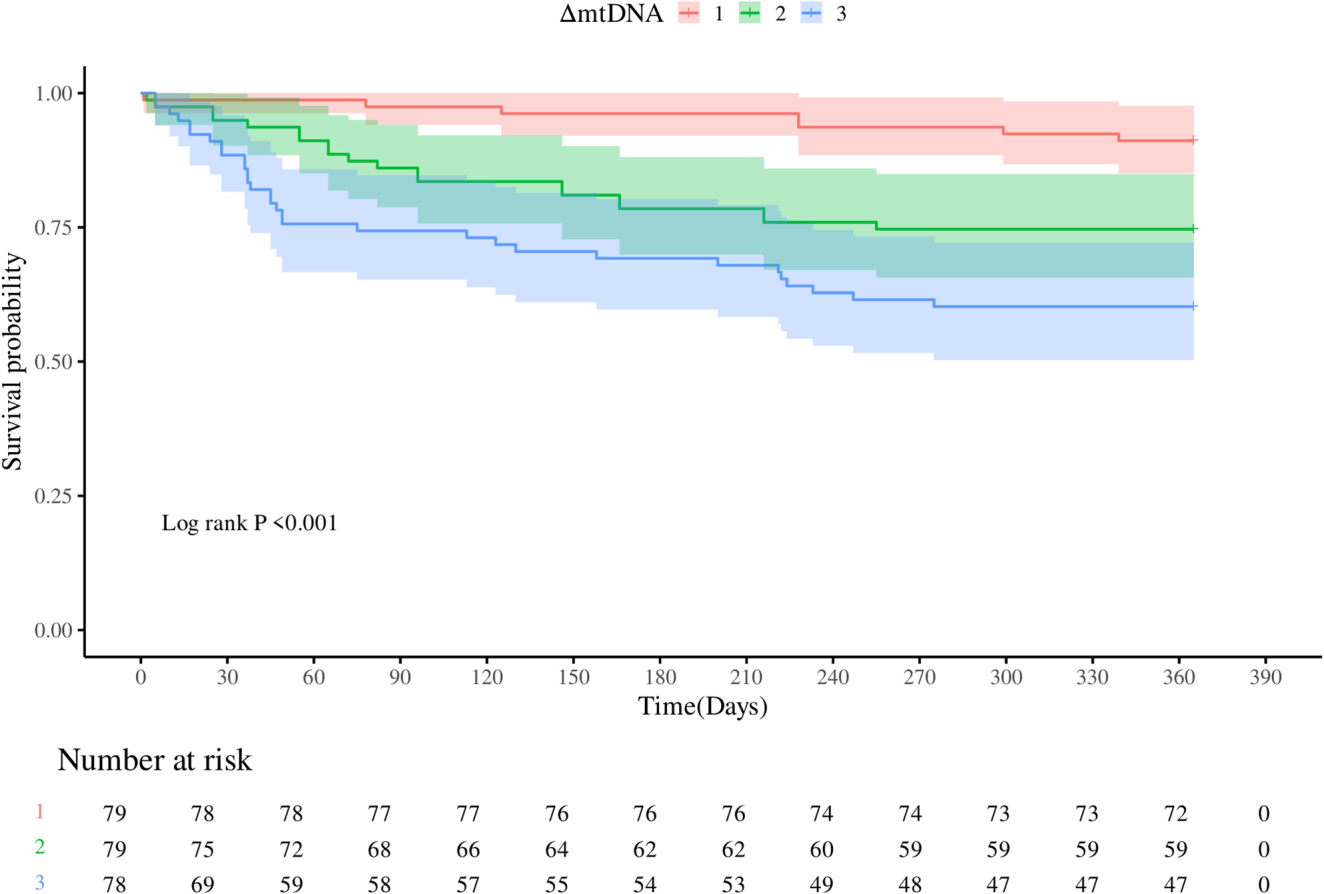
Kaplan-Meier survival curves for postoperative MACE risk in the three groups

Survival analysis using the Kaplan-Meier method assessed the relationship between ΔmtDNA levels and postoperative MACE risk. The cumulative incidence of MACE differed significantly among the three ΔmtDNA groups (Log-rank test, *P* < 0.001; Fig. 3). Analysis of individual MACE components showed that only recurrent angina incidence varied significantly by ΔmtDNA group (Log-rank test, *P* = 0.044). In contrast, all-cause mortality, non-fatal myocardial infarction, target vessel revascularization, and ischemic stroke showed no statistically significant differences across groups (Log-rank test, all *P* > 0.05; Figure S1). Therefore, the link between ΔmtDNA and the composite MACE endpoint is largely attributable to recurrent angina.

### Subgroup analysis

To evaluate the robustness of the association between ΔmtDNA and postoperative MACE risk, we performed exploratory subgroup analyses. The association between ΔmtDNA and MACE risk was consistent across subgroups stratified by sex, smoking history, comorbidities such as hypertension and diabetes, and medication categories including ACEI/ARB/ARNI and SGLT2 inhibitors. A significant interaction between ΔmtDNA and MACE risk was observed specifically in subgroups receiving β-blockers and PCSK9 inhibitors (P for interaction < 0.05). Due to the limited number of overall MACE events, these subgroup analyses and interaction findings must be considered exploratory (Supplementary Figure S2).

## Discussion

In this prospective study of STEMI patients undergoing emergency PCI, a significant positive correlation emerged between the dynamic elevation of circulating mtDNA (ΔmtDNA) during the perioperative period and the subsequent risk of MACE. This association remained significant after adjustment for key confounding factors, indicating that ΔmtDNA may reflect perioperative reperfusion injury and inflammatory status.

This study used ΔmtDNA as a core indicator to specifically capture mitochondrial stress induced by reperfusion injury. Prior work has established that mtDNA acts as a damage-associated molecular pattern, driving sterile myocardial inflammation by activating innate immune pathways such as TLR9-p38 MAPK and cGAS-STING [10, 11]. Our findings align with this framework and further support the view of Hausenloy et al. that reperfusion-induced opening of mitochondrial permeability transition pores triggers a burst release of mtDNA, which may be pivotal in microvascular dysfunction and secondary injury [12]. Although circulating mtDNA is rapidly cleared and perioperative hemodilution can reduce absolute postoperative measurements, ΔmtDNA quantifies the net release following PCI, thereby offering a more direct reflection of reperfusion injury severity. Consequently, ΔmtDNA captures the net cellular injury triggered by reperfusion more dynamically than static single time-point measurements. Its sustained elevation may drive pathological remodeling beyond the acute phase via innate immune mechanisms, potentially contributing to long-term MACE risk.

Our study found that high ΔmtDNA levels were significantly associated with recurrent angina, but not with hard endpoints like all-cause mortality or non-fatal myocardial infarction. This indicates that the overall link between ΔmtDNA and the composite MACE endpoint is largely attributable to this softer outcome. This observation aligns closely with recent research on coronary microvascular obstruction (MVO), which independently predicts adverse outcomes in STEMI patients, a process where inflammatory mediators are central to microcirculatory dysfunction [13, 14]. We speculate that elevated circulating mtDNA may directly injure vascular endothelial cells or trigger neutrophil extracellular trap (NET) formation, promoting microthrombosis and consequent persistent microvascular angina [15]. Clinically, these findings imply ΔmtDNA may serve better as a biomarker for residual ischemia risk and symptom burden after PCI than as a predictor of hard mortality endpoints.

Preoperative mtDNA levels differed significantly across the ΔmtDNA tertiles, with the highest tertile (T3) exhibiting the greatest baseline concentrations. This observation prompted an investigation into whether the prognostic value of ΔmtDNA simply reflected these baseline levels. After adjustment for preoperative mtDNA in multivariable Cox model 3, ΔmtDNA retained an independent association with MACE risk, confirming its predictive value is distinct from baseline mtDNA.

In our exploratory subgroup analysis, we observed a counterintuitive phenomenon: the statistical association between ΔmtDNA and MACE appeared stronger in patients receiving β-blockers or PCSK9 inhibitors than in untreated individuals. Pharmacologically, PCSK9 inhibitors exert anti-inflammatory and plaque-stabilizing effects beyond lipid lowering [16, 17], while β-blockers may mitigate myocardial injury by maintaining mitochondrial network homeostasis [18]. Theoretically, these protective effects should attenuate the association between mtDNA and adverse outcomes. The most plausible explanation for this paradox is the limited overall number of MACE events in this study. After stratification, the sample size and event count in the untreated subgroup were substantially reduced, resulting in severely inadequate statistical power. Consequently, this subgroup failed to detect a significant association, representing a potential false negative. In contrast, the treated group retained most samples, providing greater likelihood of demonstrating statistical significance. An alternative hypothesis invokes the ‘residual inflammatory risk’ theory: in patients where pharmacotherapy suppresses low-grade inflammatory background, elevated ΔmtDNA may reflect a resilient pathological pathway, such as persistent NLRP3 inflammasome activation, that remains refractory to treatment [19]. This could heighten the predictive specificity of ΔmtDNA. However, based on the current data, these interpretations remain speculative and do not support the conclusion that specific medications mechanistically enhance the predictive value of ΔmtDNA.

This study measured extracellular mtDNA levels in serum samples. Dhondup et al. reported that patients with chronic heart failure had significantly elevated white blood cell counts, which trended toward a correlation with plasma-free mtDNA levels [20]. Therefore, mtDNA detection in plasma samples may be confounded by factors such as leukocytes, inflammatory status, and infections. Serum was chosen to minimize contamination from intact cells, as these are removed during coagulation [21]. However, it should be noted that the clotting process itself can activate platelets, potentially leading to the release of mitochondrial contents, including mtDNA [8]. Thus, while serum avoids direct cellular contamination, it may still contain platelet-derived mtDNA released during ex vivo coagulation. This limitation should be considered when interpreting our findings. Nevertheless, circulating mtDNA can originate from various injured tissues, including endothelial cells, skeletal muscle, or liver [22]. The precise cellular origin of the detected mtDNA remains uncertain, and a contribution from non-cardiac sources cannot be excluded.

Several limitations should be acknowledged. First, the modest number of MACE events limited statistical power for complex analyses, such as restricted cubic splines or subgroup interactions, despite streamlined multivariable models. The low events-per-variable ratio may have led to overfitting and inflated effect estimates, particularly evident in the large hazard ratios from tertile analyses, which should be interpreted with caution. Second, although multivariable adjustments were performed, residual confounding cannot be ruled out, especially as key prognostic factors like total ischemic time and infarct size were unavailable. Their absence may confound the observed association between ΔmtDNA and MACE risk. Third, this single-center study lacks external validation. Importantly, due to the modest number of MACE events, we were unable to formally assess the incremental prognostic value of ΔmtDNA by comparing model discrimination (e.g., C-statistic/AUC) or reclassification indices against established clinical risk models (such as GRACE or TIMI scores). Therefore, whether ΔmtDNA provides independent predictive information beyond conventional risk factors remains to be determined. Consequently, the clinical utility of ΔmtDNA as a risk stratification tool remains preliminary and requires confirmation in larger, multicenter prospective studies. Fourth, residual confounding cannot be ruled out despite multivariable adjustments. Although we adjusted for admission Troponin T and confirmed the absence of severe multicollinearity despite its moderate correlation with ΔmtDNA, admission values are heavily influenced by ischemic time and imperfectly represent final infarct size. In the absence of systematic peak injury marker assessments or direct imaging such as cardiac magnetic resonance, we cannot exclude the possibility that the prognostic value of ΔmtDNA partly reflects a definitive necrotic burden. Fifth, the postoperative blood sampling window of 24 h is relatively broad, during which substantial time-dependent biological fluctuations in mtDNA release and clearance may occur [23]. While self-referencing via ΔmtDNA was used to mitigate inter-individual variability, sampling heterogeneity could still influence its magnitude and association with MACE, warranting serial sampling at fixed time points in future work. Finally, the detection methodology for circulating mtDNA currently lacks standardization, potentially hindering direct comparisons across studies [24].

## Conclusions

In conclusion, this exploratory study demonstrates that perioperative ΔmtDNA is associated with the risk of post-PCI MACE in STEMI patients. Notably, this association was driven primarily by recurrent angina, rather than by hard endpoints such as all-cause mortality or non-fatal myocardial infarction. These findings suggest that ΔmtDNA could function as an adjunctive biomarker to identify patients at elevated risk for ischemia-driven symptomatic events. However, the modest sample size and absence of a formal comparison with established risk scores leave the incremental prognostic value of ΔmtDNA over conventional clinical predictors uncertain. Consequently, the clinical utility of ΔmtDNA for risk stratification requires further validation in larger, multicenter studies that incorporate formal discrimination and reclassification analyses and that comprehensively assess infarct size and ischemic time.

## Supplementary Information

Supplementary Material 1

Supplementary Material 2

## Data Availability

All initial data from this study are available by e-mail to the corresponding author upon reasonable request.

## Abbreviations

ΔmtDNA: Dynamic change in circulating mitochondrial DNA (mtDNA) level
ACEI: Angiotensin-Converting Enzyme Inhibitor
ARB: Angiotensin II Receptor Blocker
ARNI: Angiotensin Receptor–Neprilysin Inhibitor
CI: Confidence Interval
CKD: Chronic Kidney Disease
CK-MB: Creatine Kinase-Myocardial Band
COPD: Chronic Obstructive Pulmonary Disease
CRP: C-Reactive Protein
cGAS-STING: Cyclic GMP-AMP Synthase–Stimulator of Interferon Genes
ECMO: Extracorporeal Membrane Oxygenation
eGFR: Estimated Glomerular Filtration Rate
HR: Hazard Ratio
IABP: Intra-Aortic Balloon Pump
IL-6: Interleukin-6
IQR: Interquartile Range
LVEF: Left Ventricular Ejection Fraction
MACEMajor: Adverse Cardiovascular Events
MAPK: Mitogen-Activated Protein Kinase
mtDNA: Mitochondrial DNA
MVO: Microvascular Obstruction
NETs: Neutrophil Extracellular Traps
NLRP3: NOD-, LRR- and Pyrin Domain-Containing Protein 3
PCI: Percutaneous Coronary Intervention
PCSK9: Proprotein Convertase Subtilisin/Kexin Type 9
PCR: Polymerase Chain Reaction
Rpm: Revolutions Per Minute
SD: Standard Deviation
SGLT2i: Sodium-Glucose Cotransporter-2 Inhibitor
STEMI: ST-Segment Elevation Myocardial Infarction
TLR9: Toll-Like Receptor 9
