# Supplementary material for: Preliminary exploration of dynamic changes in circulating mitochondrial DNA (ΔmtDNA) as a prognostic biomarker for acute ST-segment elevation myocardial infarction patients: a prospective observational study
Source: BMC Cardiovasc Disord. 2026 Jun 5;26:683. doi: 10.1186/s12872-026-06048-5 (PMC13466647; doi:10.1186/s12872-026-06048-5)
Supplement: Supplementary file 1 [file 12872_2026_6048_MOESM1_ESM.docx]

| **Table S1** Exploratory multivariable Cox regression analysis of the association between ΔmtDNA and postoperative MACE risk | | | | | | | | | | | |
| --- | --- | --- | --- | --- | --- | --- | --- | --- | --- | --- | --- |
| **mtDNA** | **Crude mode** | |  | **Model 1** | |  | **Model 2** | |  | **Model 3** | |
|  | **HR(95%CI)** | ***P* value** |  | **HR(95%CI)** | ***P* value** |  | **HR(95%CI)** | ***P* value** |  | **HR(95%CI)** | ***P* value** |
| ΔmtDNA（per 1 SD elevation） | 1.237 (1.161-1.317) | <0.001 |  | 1.237 (1.161-1.317) | <0.001 |  | 1.239 (1.161-1.321) | <0.001 |  | 1.336 (1.195-1.494) | <0.001 |

Crude model was adjusted for none

Model 1 was adjusted for age

Model 2 was adjusted for model 1 + (estimated glomerular filtration rate, left ventricular ejection fraction, Killip class)

Model 3 was adjusted for model 1 + model 2 + (Troponin T, culprit vessel, access site, multivessel disease, mechanical circulatory support, preoperative mtDNA)

**Table S2** Exploratory analysis: Association between ΔmtDNA categories (tertiles) and postoperative MACE risk

| **mtDNA** | **Crude mode** | |  | **Model 1** | |  | **Model 2** | |  | **Model 3** | |
| --- | --- | --- | --- | --- | --- | --- | --- | --- | --- | --- | --- |
|  | **HR(95%CI)** | ***P* value** |  | **HR(95%CI)** | ***P* value** |  | **HR(95%CI)** | ***P* value** |  | **HR(95%CI)** | ***P* value** |
| T1 | 1 |  |  | 1 |  |  | 1 |  |  | 1 |  |
| T2 | 3.190(1.349-7.546) | 0.008 |  | 3.190(1.349-7.546) | 0.008 |  | 3.194(1.344-7.588) | 0.009 |  | 3.008(1.235-7.328) | 0.015 |
| T3  P for trend | 5.654(2.488-12.849)  <0.001 | <0.001 |  | 5.654(2.488-12.849)  <0.001 | <0.001 |  | 6.141(2.636-14.306)  <0.001 | <0.001 |  | 3.600(1.248-10.389)  <0.001 | 0.018 |

Crude model was adjusted for none

Model 1 was adjusted for age

Model 2 was adjusted for model 1 + (estimated glomerular filtration rate, left ventricular ejection fraction, Killip class)

Model 3 was adjusted for model 1 + model 2 + (Troponin T, culprit vessel, access site, multivessel disease, mechanical circulatory support, preoperative mtDNA)
