# Supplementary material for: Preliminary exploration of dynamic changes in circulating mitochondrial DNA (ΔmtDNA) as a prognostic biomarker for acute ST-segment elevation myocardial infarction patients: a prospective observational study
Source: BMC Cardiovasc Disord. 2026 Jun 5;26:683. doi: 10.1186/s12872-026-06048-5 (PMC13466647; doi:10.1186/s12872-026-06048-5)
Supplement: Supplementary file 2 [file 12872_2026_6048_MOESM2_ESM.docx]

A B

C D

**Fig. S1** (A) Kaplan-Meier survival curves for postoperative all-cause mortality in the three groups. (B) Kaplan-Meier survival curves for postoperative non-fatal myocardial infarction in the three groups. (C) Kaplan-Meier survival curves for freedom from postoperative target vessel revascularization in the three groups. (D) Kaplan-Meier survival curves for freedom from postoperative recurrent angina in the three groups

**Fig. S2** Exploratory subgroup analysis and interaction tests for the association of ΔmtDNA with MACE risk
